# Changes in the root-associated bacteria of sorghum are driven by the combined effects of salt and sorghum development

**DOI:** 10.1186/s40793-021-00383-0

**Published:** 2021-08-11

**Authors:** Gao Yukun, Cui Jianghui, Ren Genzeng, Wei Shilin, Yang Puyuan, Yin Congpei, Liang Hongkai, Chang Jinhua

**Affiliations:** College of Agronomy, Hebei Agricultural University, Northern China Key Laboratory for Crop Germplasm Resources of Education Ministry, No. 2596 LeKai South Street, Baoding, Hebei China

**Keywords:** Rhizosphere soil, Bacterial community diversity, Sorghum, Salinity

## Abstract

**Background:**

Sorghum is an important food staple in the developing world, with the capacity to grow under severe conditions such as salinity, drought, and a limited nutrient supply. As a serious environmental stress, soil salinization can change the composition of rhizosphere soil bacterial communities and induce a series of harm to crops. And the change of rhizospheric microbes play an important role in the response of plants to salt stress. However, the effect of salt stress on the root bacteria of sorghum and interactions between bacteria and sorghum remains poorly understood.

**Results:**

The purpose of this study was to assess the effect of salt stress on sorghum growth performance and rhizosphere bacterial community structure. Statistical analysis confirmed that low high concentration stress depressed sorghum growth. Further taxonomic analysis revealed that the bacterial community predominantly consisted of phyla *Proteobacteria*, *Actinobacteria*, *Acidobacteria*, *Chloroflexi*, *Bacteroidetes* and *Firmicutes* in sorghum rhizosphere soil. Low salt stress suppressed the development of bacterial diversity less than high salt stress in both bulk soil and planted sorghum soil. Different sorghum development stages in soils with different salt concentrations enriched distinctly different members of the root bacteria. No obviously different effect on bacterial diversity were tested by PERMANOVA analysis between different varieties, but interactions between salt and growth and between salt and variety were detected. The roots of sorghum exuded phenolic compounds that differed among the different varieties and had a significant relationship with rhizospheric bacterial diversity. These results demonstrated that salt and sorghum planting play important roles in restructuring the bacteria in rhizospheric soil. Salinity and sorghum variety interacted to affect bacterial diversity.

**Conclusions:**

In this paper, we found that salt variability and planting are key factors in shifting bacterial diversity and community. In comparison to bulk soils, soils under planting sorghum with different salt stress levels had a characteristic bacterial environment. Salinity and sorghum variety interacted to affect bacterial diversity. Different sorghum variety with different salt tolerance levels had different responses to salt stress by regulating root exudation. Soil bacterial community responses to salinity and exotic plants could potentially impact the microenvironment to help plants overcome external stressors and promote sorghum growth. While this study observed bacterial responses to combined effects of salt and sorghum development, future studies are needed to understand the interaction among bacteria communities, salinity, and sorghum growth.

**Supplementary Information:**

The online version contains supplementary material available at 10.1186/s40793-021-00383-0.

## Background

Salt stress is one of the most significant obstacles to agricultural productivity worldwide, arid and semi-arid climate zones are the most affected. Salinity affects abscisic acid synthesis, which decreases photosynthesis and causes oxidative stress, osmotic stress, ion toxicity, and mineral deficiencies, leading to inhibition of plant growth and eventually decreasing production [1–3]. Additionally, salinity affects soil biodiversity, microbial activities which alters the soil physicochemical properties, leading to organic matter reduction and sodification [4]. Over ~ 6% of the world’s lands are saline and ~ 30% of irrigated lands are suffering from salinity problems in the world [5]. To cope with environmental stress, plants develop strategies that include changes in growth activities, such as lowering rates of photosynthesis, decreasing transpiration, changing the root system structure and function, and activating cascades of molecular networks involved in stress sensing, signal transduction, and the expression of specific stress-related genes and metabolites [6–8]. These changes could shift the rhizospheric soil microbial community to increase the plant root uptake capability under stress [9]. The rhizosphere microbial community structure is the result of a complex series of interactions and feedback loops among plant roots, microorganisms and the physical and chemical environment of the soil [10–16]. Within the rhizosphere, complex and dynamic interactions occur between plants and microorganisms. Distinct rhizosphere microbiomes have been shown to exist near the roots of different species of plants [17]. Moreover, plant-associated microbiomes play an important role in allowing plants to adapt to environmental factors and other specific traits [18, 19]. The microbiome can increase plant productivity by secreting hormones and improving nutrient availability of plants, and plants can recruit management-system-specific taxa for rhizosphere [20, 21]. Geddes et al. [22] constructed a synthetic signal network between plants and bacteria to regulate the expression of bacterial genes in the rhizosphere, which promoted the growth and development of plants.

Similarly, rhizospheric microbes play important roles in plant growth under salt stress. Numerous studies have demonstrated the direct relationships of rhizospheric microbial community structure and diversity with soil salt [23–26]. Some studies have reported that microbial diversity linearly decreases with increases in salinity and that community dissimilarity significantly increases with the salinity difference [27]. For example, a high concentration of salt affects the production of legume nodules [28, 29]. The research had found that *Pseudomonas syringae* and *Pseudomonas fluorescens* show increased plant growth and yield under salt stress and increased leaf chlorophyll content. *Rhizobium* and *Pseudomonas* can promote the biosynthesis of proline and maintain the relative moisture content of leaves and the selective absorption of K^+^ ions. *Pseudomonas putida* can reduce the plant’s intake of Na^+^ and increase the absorption of Mg ^2+^, K^+^, and Ca^2+^ [26].

Sorghum (*Sorghum bicolor*) is the fifth most important creeal crop worldwide. It is considered to be moderately tolerant to salt, particularly more tolerant than maize [30]. It is an important food staple in the developing world and dry and relatively more saline regions. Because of the innate nature of tolerance to salinity stresses, sorghum is considered a model system for studying plant salt responses among cereals [31]. Sorghum has been widely cultivated for food and animal feed in tropical and subtropical regions and, in particular, in marginal areas including saline soils for its high abiotic tolerance. Sorghum could tolerate soil and water salinity up to 6.8 and 4.5 dS m^−1^of electrical conductivity, respectively [4].

The phenotypic impact of salinity on sorghum can be impactful, depending on the variety, developmental stage and salt concentration. Upon sensing soil salinity, sorghum activated transcriptional changes enabling them to deploy mechanisms for adaptation to salt stress, and redirection of growth patterns. The plant signaling events underpinning the adaptive responses to salt stress complex and plant physiological and biochemistry pathways and soil microbe shifts was involved [3, 5, 9]. It has been reported that drought shifts the microbe diversity and community of sorghum roots and depletes the expression of genes critical to arbuscular mycorrhizal (AM) symbiosis, with a corresponding drop in AM fungal mass in the plant roots [32]. However, little is known about the pattern of variation in the root microbiota in sorghum under salt stress. Another study had shown that AM could help to alleviate the negative effects caused by salinity, and showed potential in biomass production of sweet sorghum in saline soil [33].

Our previous study revealed that salt stress exerted different effects on gene expression in different salt-tolerant sorghum genotypes. This transcriptomic response includes genes that have critical functions in biotic defense and abiotic stress responses [34]. In this study, we selected the high salt-tolerant variety GLZ and the salt-sensitive variety HN16 to examine the composition and variation in root bacteria under salt stress to identify links among root bacteria, sorghum populations, and salt stress tolerance, providing reference to increase sorghum salt tolerance and use saline soil.

## Results

### Effects of salt stress on sorghum phenotypic traits

When a plant is under stress, the plant height, roots and yield are important features that reflects the level of stress [34]. To evaluate the salt tolerances of the two sorghum varieties in this experiment, their plant heights and panicle traits at the time of ripening were investigated. The results showed that the plant height of GLZ increased significantly under low salt stress, reaching 12.99% higher than the control, and HN16 was 4.19% lower than the control (Table 1). Under high salt stress, the plant height of the two cultivars showed a significant decrease compared with the control. GLZ and HN16 height decreased 16.77 and 23.84% compared with the control, respectively.

**Table 1:** The effect of salt stress on sorghum plant height and panicle traits

Salinity	GLZ				HN16			
	S0	S3	S5	S7	S0	S3	S5	S7
PH(cm)	164.0 ± 5.6b	185.3 ± 5.12a	146.7 ± 4.2c	136.5 ± 3.7d	90.6 ± 5.2a	86.8 ± 3.4ab	82.1 ± 2.54b	69.0 ± 4.0c
MPL (cm)	11.60 ± 1.51a	9.76 ± 0.26a	9.47 ± 3.78a	9.17 ± 1.44a	23.87 ± 1.34a	20.93 ± 4.07ab	20.83 ± 0.53ab	17.77 ± 2.40b
MPW (g)	8.73 ± 1.06a	6.27 ± 0.15b	5.92 ± 0.36b	5.20 ± 0.70b	7.73 ± 0.23a	7.14 ± 0.67a	5.47 ± 0.67b	4.27 ± 0.21c
TGW (g)	15.43 ± 0.47c	16.48 ± 0.43c	18.33 ± 1.36b	20.63 ± 1.10a	22.97 ± 0.21b	27.77 ± 0.42a	28.03 ± 0.80a	29.87 ± 0.31a

Values are means±SD; Means with the same letter in the column are not significantly different in same variety (*p* > 0.05)

We also measured the main panicle length and weight of the two varieties under salt stress (Table 1). The results showed that salt stress led to the decrease of main panicle length (MPL) and weight (MPW) of sorghum, and the decrease reached the largest under S7 treatment. Compared with the control, the main panicle length and weight of GLZ decreased by 20.95 and 40.44%, respectively, and HN16 were 25.56% and 44.76%, respectively. While, the thousand grain weight (TGW) increased by 33.70 and 30.04% respectively in GLZ and HN16 under salt treatment compared with the control.

The results showed sorghum grew well under low salt stress, and the growth was inhibited by high salt stress. Variety GLZ had better tolerance to salt than HN16.

### Overall bacterial 16S rRNA gene sequencing results

To explore the effects of salt and sorghum development on root-associated bacterial communities, the pot culture experiment was conducted in the greenhouse. Sorghum plant rhizosphere soils were sampled for bacterial-related traits analysis.

A bacterial community profile for each sample was generated using Illumina MiSeq sequencing of the V3–V4 region of the 16S rRNA gene. After quality filtering of the raw reads and exclusion of non-target sequences, a total of 2,967,612 high-quality sequences with 20,427–45,283 sequences and 8493 OTUs with 2544–3805 OTUs were obtained for each sample (*n* = 84, Table S1). The dominant phyla (average relative abundance > 1%) were *Proteobacteria* (30.50%), *Actinobacteria* (18.61%), *Acidobacteria* (15.18%), *Chloroflexi* (9.17%), *Bacteroidetes* (8.87%), *Firmicutes* (5.19%), *Gemmatimonadetes* (4.29%), *Patescibacteria* (2.06%), *Verrucomicrobia* (1.49%), *Planctomycetes* (1.37%) and *Cyanobacteria* (1.13%). *Proteobacteria* was the most abundant phylum (Fig. S1). All the calculated diversity indices in all samples (*n* = 84), including sobs, Shannon, and Chao, are shown in Table 2. PERMANOVA was performed using the Bray-Curtis distances for samples. This analysis revealed that bacterial communities were strongly affected by salt, followed by growth and variety. Interactions between salt and growth and between salt and variety were also detected (Table 3).

**Table 2:** Average OTUs diversity with different treatments

Sample code	sobs	Shannon	Simpson	ace	bootstrap	chao
1	3305.6667	6.52524	0.007885	4700.241087	3842.435531	4749.283604
2	3471	6.916164	0.002518	4744.123419	3991.881742	4808.416643
3	3687.6667	6.865832	0.002944	5098.106027	4248.530173	5082.931561
4	2630.6667	5.894435	0.012427	4195.693083	3055.376641	3829.909156
5	3123	6.459669	0.010222	4391.73295	3613.195989	4409.180985
6	2999	6.584501	0.003932	4209.685494	3460.999989	4260.658434
7	3011.6667	6.523709	0.004566	4240.668224	3483.474087	4273.250191
8	3015.6667	6.446661	0.006265	4253.034677	3489.817222	4256.609971
9	3008.3333	6.645542	0.004507	4279.648553	3486.157715	4332.155195
10	2939.6667	6.516377	0.005073	4319.749604	3389.531527	4166.014243
11	2835.6667	6.623655	0.003435	4234.392741	3279.303407	4031.106482
12	2728.3333	6.393818	0.005552	4078.79015	3159.361593	3908.638395
13	3310.3333	6.825167	0.003395	4723.665437	3846.348963	4728.4829
14	3096	6.620117	0.006291	4343.143509	3579.669837	4379.002823
15	3575.6667	6.890484	0.002935	4987.491659	4124.877401	5017.769223
16	3019.3333	6.474631	0.004986	4315.02025	3498.7692	4371.396951
17	3034	6.644153	0.004401	4285.130032	3518.859683	4300.025573
18	2914.3333	6.748933	0.002972	4306.798259	3371.78901	4168.089047
19	2891.6667	6.485932	0.004442	4412.309759	3355.833095	4154.814489
20	3093.6667	6.534835	0.004937	4331.856336	3568.962295	4318.329487
21	2919.3333	6.574735	0.004923	4425.62505	3392.668895	4226.117628
22	2925	6.39938	0.006546	4055.038863	3373.554613	4069.441289
23	3175.3333	6.371499	0.00825	4450.180355	3670.903476	4460.348312
24	3170.6667	6.60916	0.003632	4633.611128	3652.243474	4472.420654
25	2871	6.300442	0.007127	4226.194975	3307.740379	4019.458144
26	2850	6.473703	0.006271	3891.236407	3271.032293	3894.491813
27	3213.6667	6.700299	0.003865	4446.86707	3698.081637	4492.786441
28	2931	6.514895	0.005496	3988.600961	3365.752284	4074.951136

**Table 3:** PERMANOVA analysis

Source of variance	Df	Sums Of Sqs	Mean Sqs	F.Model	R^2^	Pr (>F)
Variety	1	0.00874	0.00874	0.6446	0.0063	0.652
Salt	3	0.414	0.138	10.1791	0.2982	0.001***
Growth	2	0.16742	0.08371	7.5545	0.12059	0.001***
Variety×Salt	3	0.09792	0.032639	2.4075	0.07053	0.012*
Growth×Salt	6	0.14205	0.023675	2.1366	0.10232	0.004**

*, **, *** represent *p* < 5, 1%, 0.1% respectively

### Effects of planting sorghum on bacterial diversity

**Fig. 1:**
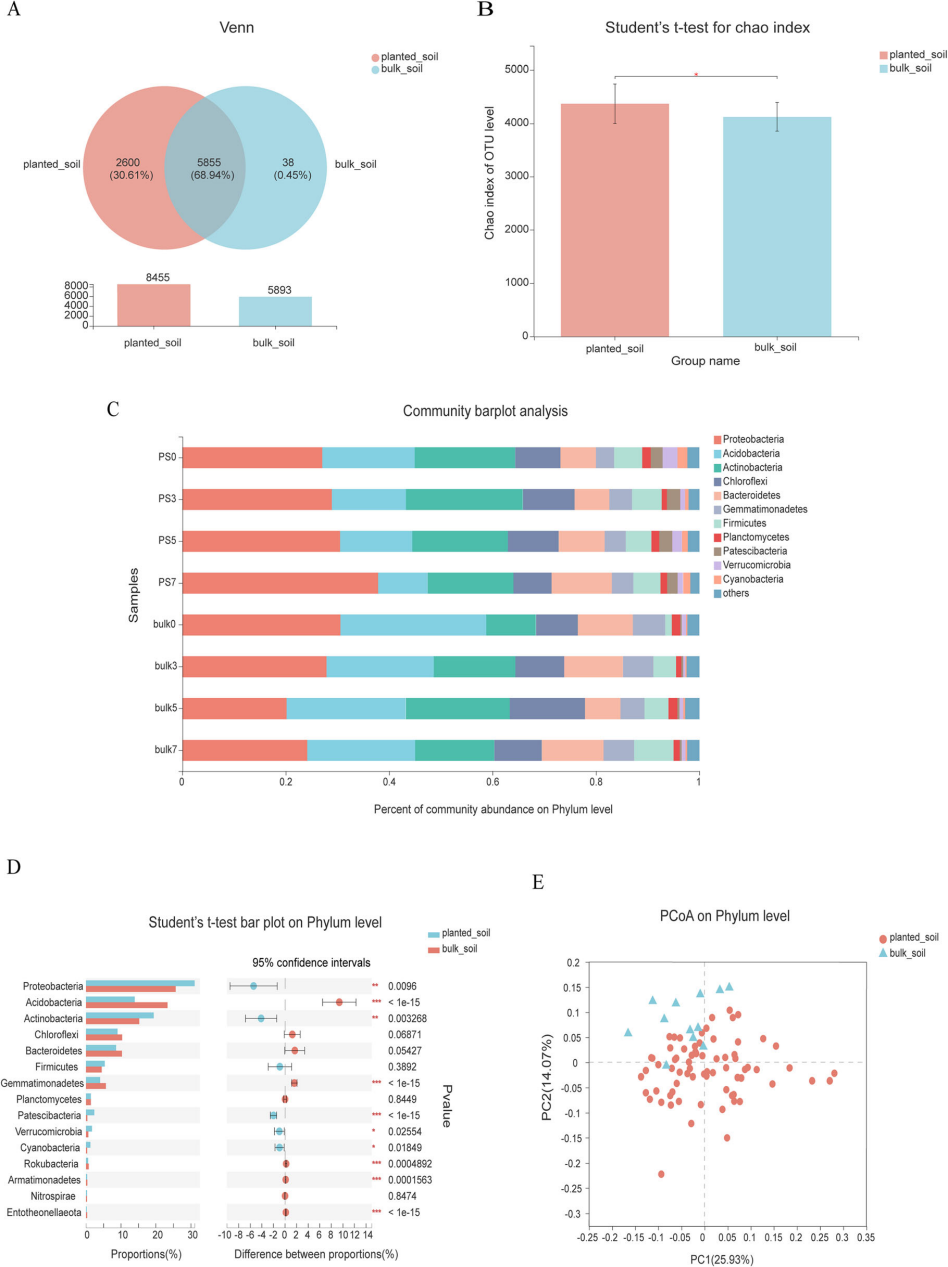
The effects of planting sorghum on bacterial diversity. **A** Commcon and specific OTUs for bulk soil and rhizosphere soils (planted soil). **B** The *t*-test showing the significant difference of diversity index between bulk soil and rhizosphere soils (planted soil) (*p* < 0.01). **C** Phylum-level distribution of the bulk soil and planted soil bacteria. **D** Phylum-level distribution and differences between bulk and rhizosphere soils (planted soil). **E** Unconstrained PCoA with Bray-Curtis distance showing the separation of planted soil and bulk soil bacteria (*p* < 0.001, PERMANOVA by Adonis)

We first compared the bulk soil and sorghum rhizosphere soil to evaluate the effect of planting sorghum on the bacterial community. A total of 8455 OTUs belonging to 999 genera in rhizosphere soil and 5893 OTUs belonging to 799 genera in bulk soils were obtained. There were 5855 OTUs overlapping between planted and bulk soils, and 2600 and 38 OTUs specific for planted and bulk soils, respectively (Fig. 1A), showing that the bacterial community underwent a significant shift after sorghum was planted, 2562 OTUs more in planting soil than that in the bulk soils. We explored the relationship between bacteria within-sample diversity (α-diversity) (observed OTUs) and planting sorghum. Measurement of the α-diversity showed a significant difference between rhizosphere soil and bulk soil at the OTU level (Fig. 1B). At the OTU level, in planting sorghum soil, the bacteria α-diversity was higher than that in bulk soil. The relative abundances of *Acidobacteria* (23.17%), *Gemmatimonadetes* (5.64%), *Rokubacteria* (0.75%), *Armatimonadetes* (0.40%) and *Entotheonellaeota* (0.31%) were higher in bulk soil than that in rhizosphere soil of sorghum, whereas *Actinobacteria* (19.38%), *Patescibacteria* (2.37%), *Verrucomicrobia* (1.65%), and *Cyanobacteria* (1.26%) were more abundant in sorghum-planted soil in the first 10 phyla (Fig. 1C, D). Unconstrained principal coordinate analysis (PCoA) of the Bray-Curtis distances demonstrated that the bacteria in the rhizosphere soil and bulk soil formed two distinct clusters that were separated along the first coordinate axis (Fig. 1E), indicating that the largest source of variation in the soil bacteria was sorghum planting. Planting sorghum significantly changed the bacterial community and diversity either in saline soil or control.

### Root bacteria and salt content

**Fig. 2:**
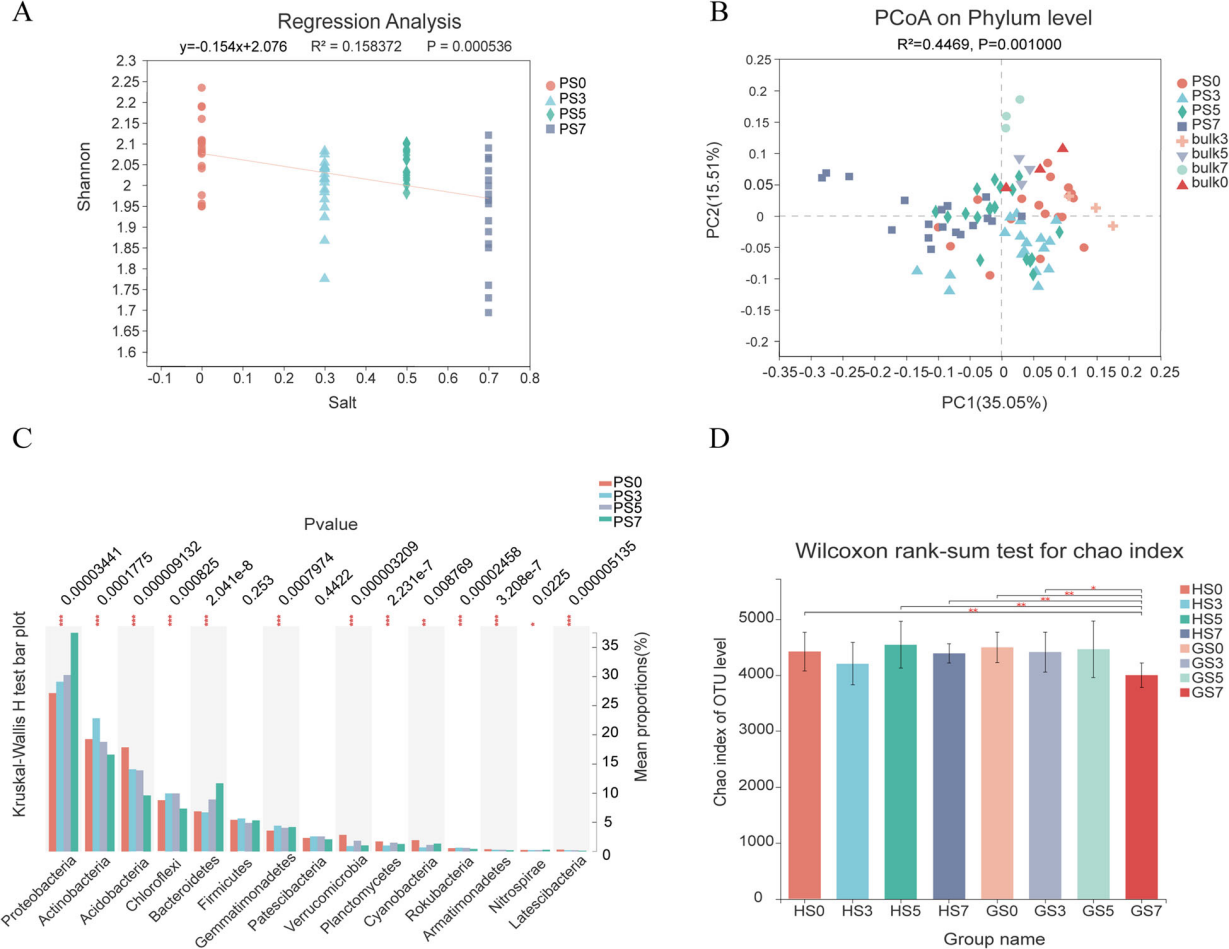
The relationship of the root bacteria and salt concentration. **A** Environmental correlation analysis showed that salinity decreased the bacterial Shannon diversity in rhizosphere soils (PS 0, PS 3, PS 5, and PS 7 represent rhizosphere soils under 0, 0.3, 0.5, and 0.7% salt concentration treatments, respectively). **B** Unconstrained PCoA (for principal coordinates Pco1 and Pco2) with a Bray-Curtis distance showing the root bacteria separated by the salt concentration in bulk soil (Bulk 0, Bulk 3, Bulk 5, and Bulk 7 represent bulk soil under 0, 0.3, 0.5, and 0.7% salt concentration treatment, respectively) and planted soil (PS0, PS3, PS5 and PS7) in the first axis (*p* < 0.001, permutational multivariate analysis of variance (PERMANOVA) by Adonis. **C** Phylum-level differences in bacteria among different salt concentrations in rhizosphere soils. **D** Salt stress had different effects on the high salt tolerance variety and sensitive variety (GS0, GS3, GS5 GS7, HS0, HS3, HS5, and HS7 represent variety GLZ(G) and HN16(H) under 0, 0.3, 0.5, and 0.7% salt concentration treatment respectively)

To explore the impact of salt on the development of the rhizosphere soil bacteria, the differences in bacteria were examined as a function of soil salt content at the OTU level. Soil salinity showed a strong effect on the bacterial community dissimilarity in both bulk and planted soils. Salinity decreased the bacteria Shannon diversity in rhizosphere soil (Fig. 2A). Principal coordinate analysis (PCoA) of the Bray-Curtis distances demonstrated that samples fell into four groups according to the salt concentration in rhizosphere soil and bulk soil, which showed that salinity stress drove bacterial diversity changes (Fig. 2B). However, unlike bulk soil, there was overlap among different salt concentrations in rhizosphere soil, showing the effect of sorghum planting on the bacteria diversity. The compositional undulation of the rhizosphere bacteria in soils with different salt contents was caused by significant changes in the relative abundances of dominant phyla, including *Proteobacteria*, *Firmicutes*, *Actinobacteria*, *Acidobacteria*, *Chloroflexi*, *Bacteroidetes*, and *Gemmatimonadetes* (Fig. 2C). In rhizosphere soil, the relative abundances of *Proteobacteria* and *Bacteroidetes* increased significantly with increasing salt concentration, and *Acidobacteria* and *Latescibacteria* decreased. The abundances of *Actinobacteria* and *Chloroflexi* reached their highest levels at S3, indicating that low salt stress benefited the development of these bacteria.

Even though PERMANOVA showed no difference betweenthe different varieties, an interaction was detected. Within-sample diversity (α-diversity) based on OTU relative abundances revealed that salt stress had different effects on root bacterial diversity in the different sorghum varieties. In the high-tolerant genotype (GLZ), high salt stress (S7) showed a great effect on the OTU relative abundances, but no significant effect was observed for the salt-sensitive variety HN16, revealing that high salt content had different effects on bacterial abundances in different varieties (Fig. 2D).

**Fig. 3:**
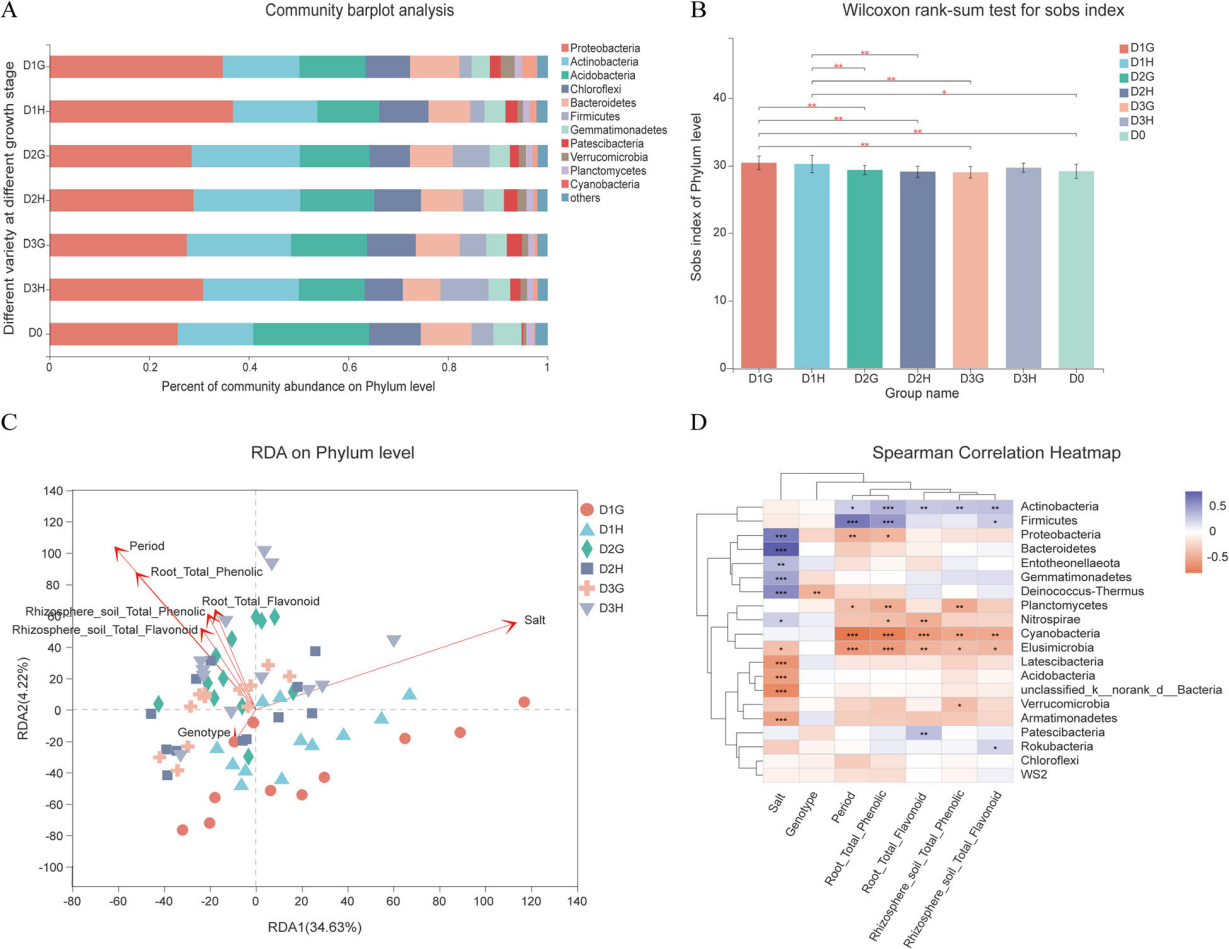
Bacterial diversity driven by the sorghum growth stage. **A** The effect of sorghum development on bacterial community is different in high-tolerance and sensitive genotypes (D0,D1,D2,D3 represent initial, elongation, flowering, and maturity time respectively). **B** The bacterial diversity showed differences at different development stages. **C** Distance-based redundancy analysis showed the effect of environmental factors on bacterial community. **D** The correlation between environmental factors and bacteria. Note: *, **, *** represent *p* < 0.05, 0.01, 0.001 respectively

### Bacterial diversity is driven by sorghum growth stage

In different growth periods, shifts in bacterial diversity were detected in the two varieties (Fig. 3A). The comparison showed that sorghum development had a significant effect on bacterial composition at phylum level (Fig. 3B). Phylum-level relative abundances plots revealed that rhizosphere bacterial communities shifted from the initial period (D0) to stages D1 to D3. The result showed that the effect of plant growth on bacterial communities was more obvious at the sorghum vegetative growth stage, and bacterial abundances relative stability after flowering (from D2 to D3) (Fig. 3B) *Proteobacteria* and *Cyanobacteria* were dramatically higher in the jointing stage than in the other two stages. However, the different varieties showed different interactions with the bacteria. For the sensitive variety HN16, the influence of growth on bacterial diversity was less than that of the tolerant variety GLZ (Fig. 3B). Constrained analysis of communities against development time using distance-based redundancy analysis (RDA) also revealed a clear gradual community shift across the different development time points for rhizosphere bacteria, and the shifts correlated with the changes in the soil salt content and sorghum variety (Fig. 3C), indicating complex interactions among the soil salt content, growth stage, and plant genotype. The strongest correlations for the bacterial community were with the soil salt content, followed by sorghum development (Fig. 3D). These analyses revealed that at different sorghum development times, salt stress had different effects on bacterial diversity and community.

### Root bacteria as an indicator for salt concentration in sorghum-planted soil

**Fig. 4:**
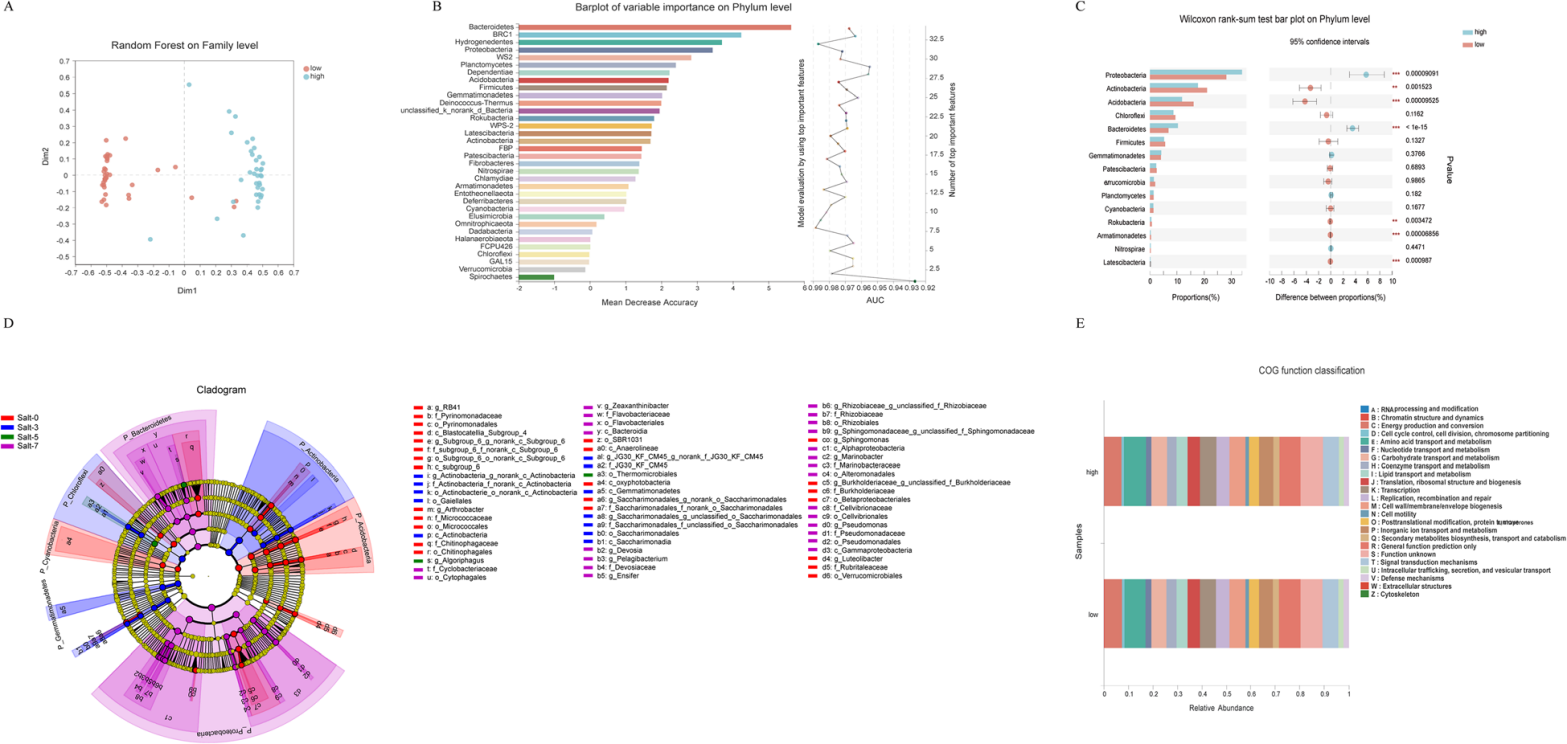
Different effects of low and high salt stress on bacterial families. **A** The samples fell into two different group based on low and high salt stress by random forest analysis. **B** The top 20 bacterial phyla were identified by applying random forest classification of the relative abundance of the root bacterial under high and low salt stress. Biomarker taxa are ranked in descending order of importance to the accuracy of the model. The curve represents validation as a function of the number of input phyla used to differentiate high and low salt root bacteria in order of variable importance. **C** Phylum-level differences of bacterial between high salt and low salt concentrations in rhizosphere soils. **D** Different colored nodes represent the bacteria groups that were significantly enriched in the corresponding groups and had a significant influence on the differences between groups; light yellow nodes represent the bacteria groups that had no significant difference in different groups or no significant influence on the differences between groups (salt 0, salt 3, salt 5, and salt 7 represent rhizosphere soil under 0, 0.3, 0.5, and 0.7% salt concentration treatment, respectively). **E** COG function prediction of root bacteria in high salt and low salt rhizosphere soil

Based on the results, we found that sorghum grew well under low salt and that sorghum growth was suppressed by high salt concentrations (≥0.5%) (Table 1). We divided the salt treatments into two classes (high salt stress, S5 & S7, and low salt stress, S0 & S3). According to this grouping, the samples fell into two significantly different groups (Fig. 4A). We further established a model using random forest to correlate high salt and low salt stress with the root bacteria data at the phylum level, and we evaluated the accuracy of root bacteria classification (Fig. 4B). The top 20 bacterial phyla were identified by applying random forest classification of the relative abundance of the root bacteria in the two groups. In relation to high and low salt stress, the results using bacterial phyla showed that the AUC value was more than 92%, indicating high accuracy (Fig. 4B). We compared the differences among all the important phyla using the Wilcoxon rank-sum test (Fig. 4C). Of the 15 most important phyla, 2 showed higher relative abundances in high salt than at low salt, while 5 phyla showed higher relative abundances in low salt. These results were consistent with the observation that low-salinity rhizosphere soil had higher bacterial diversity (Fig. 2A). LEfSe analysis indicated that the bacteria responded differently to high and low salt stresses at different taxonomic levels (Fig. 4D, Fig. S2). Functional prediction further showed that many members of the bacteria were involved in substance transport and metabolism, indicating that these members play crucial roles in supporting sorghum survival under salt stress (Fig. 4E). These bacteria can be as indicators for the salt concentration in sorghum planting field.

### Analysis of total phenol and total flavonoid contents in sorghum root and rhizosphere soil during sorghum development

**Fig. 5:**
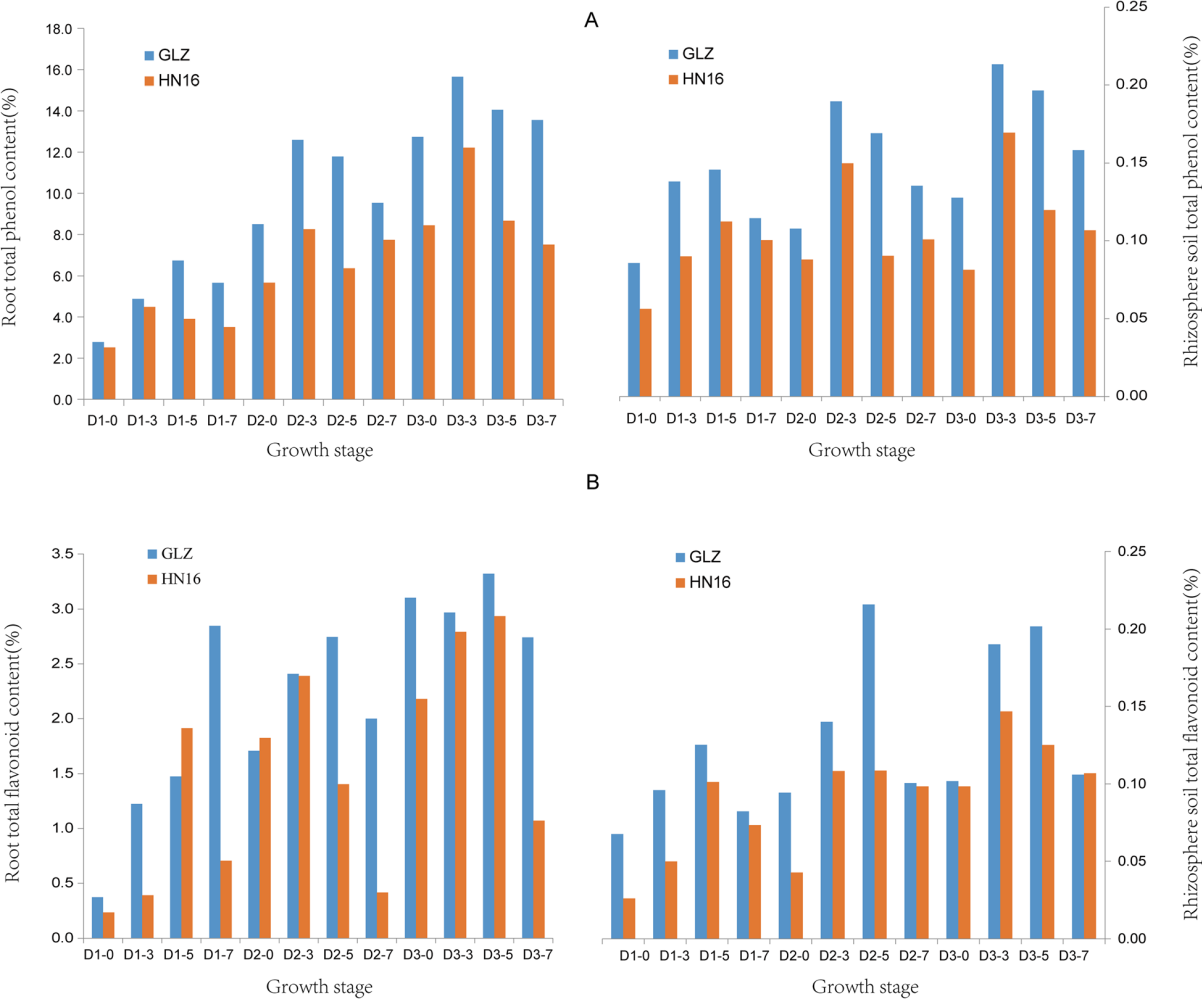
Differences in total phenol and total flavonoid contents in sorghum root and rhizosphere soil during sorghum development. **A** Total phenol content in sorghum root and rhizosphere soil during sorghum development in different varieties. **B**. Total flavonoid content in sorghum root and rhizosphere soil during sorghum development in different varieties

**Fig. 6:**
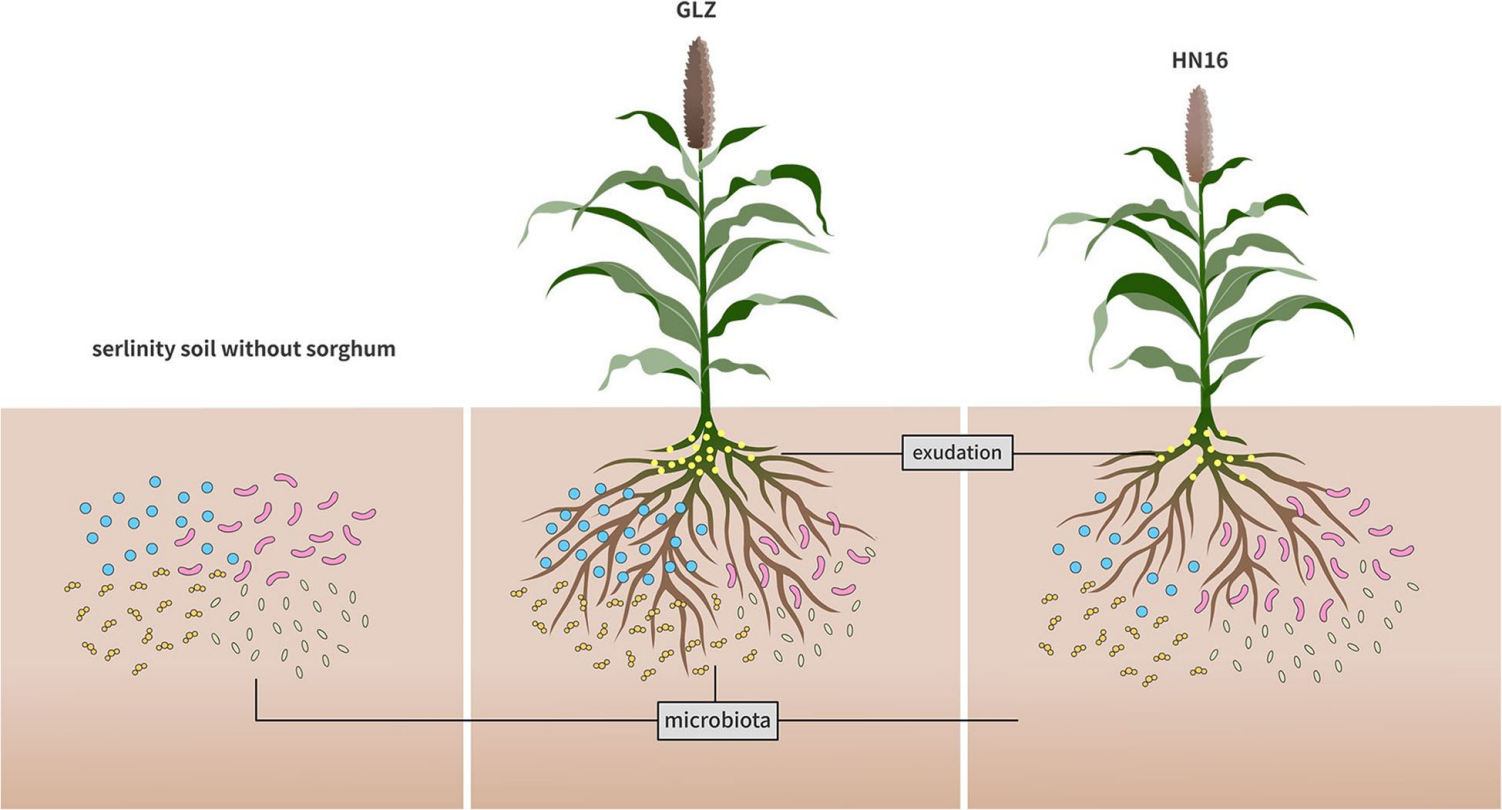
The interaction model of sorghum and salinity influenced the root exudation process and thereby changed the rhizobacterial community

Plants can exude variable substances to adapt to their environment. Phenolic compounds are present in all sorghums and are important antioxidants. To determine their functions in the resistance to salt stress and whether they influence the soil bacteria, we analyzed the total phenol and total flavonoid content in the roots and rhizosphere soil of different sorghum varieties at different developmental stages under different salt stresses. The results showed that the total phenol and total flavonoid contents had a positive correlation between the root and root soil (*r* = 0.824, 0.671, *p* < 1%, Table S2), and the total phenol content was highest either in sorghum roots or in root soil at 0.3% salt content and creased with sorghum development (D2 & D3). The total phenol content in sorghum roots and the rhizosphere soil decreased with increasing salt content in the soil but was higher than that in the control (D1 & D2) (Fig. 5). Moreover, the total phenol content in GLZ was higher than that in HN16 in both roots and root soil. The shifts in total flavonoid content in roots and rhizosphere soil were slightly different from the shifts in phenol content. At high salt stress (S7), the flavonoid content was higher in GLZ roots than in HN16, but in root soil, there was no difference between the two varieties. An analysis of the relationships among the total phenol and flavonoid contents and bacterial community, variety, and development revealed that phenolic compounds had a significant effect on bacterial diversity and interacted with sorghum development, salt concentration, and variety (Fig. 3D). The above results indicated that phenolic compounds play a special role in sorghum salt tolerance. Sorghum could adjust its fitness to salt by shifting phenolic compound biosynthesis (Fig. 6).

## Discussion

Agricultural productivity worldwide is subject to environmental constraints, particularly drought and salinity, due to their wide distribution. Sorghum is one of the most tolerant plants to salinity stresses, and understanding how it copes with stress is the first step in developing germplasms with good fitness under salinity stress. Plant-associated microbes can compete with the plant and with each other for nutrients but may also carry traits that increase the productivity of the plant [35]. During their growth, plants will continue to interact with rhizosphere microorganisms to adapt to their habitats. These microorganisms are called plant growth-promoting rhizobacteria (PGPR) [36–39]. For example, an increase in the abundance of bacteria of *Actinobacteria* was reported in rhizosphere soil of plants during late developmental stages [40]. Studies have shown that plants regulate their root exudates by identifying volatile substances released by microbes in their rhizosphere and that this stimulates nutrient absorption and defense signaling pathways [37, 41].

This study analyzed the effect of salinity and sorghum genotype on the bacterial community diversity and structure. We found that when planting sorghum, salinity and plant development had pronounced effects on the bacterial community and diversity (Fig. 3A). Comparing the effects of salinity, planting sorghum, plant growth, and genotype showed that salinity, planting, and plant growth greatly contributed to bacterial restructuring (Fig. 3A), which may have been caused by root system activity, salt and plant growth interactions, and competition between plant-associated microbes and plant growth, which induced changes in soil nutrients and soil microecology [13, 14, 42].

The results of this study support that salt stress was one of the most important factors affecting both bacterial community diversity and structure (Tables 2, 3, Fig. 2). The soil salt content is thought to be an important factor that changes the soil osmotic pressure, leading to water loss from microbial cells, which inhibits bacterial growth and even causes death, leading to alterations in microbial community structure; conversely, bacteria can adjust the plant’s ability to adapt to salt stress [31, 43]. In this study, the detected bacterial taxa belonging to *Proteobacteria*, *Acidobacteria*, *Actinobacteria*, *Rokubacteria*, and *Armatimonadetes* were significantly different between high- and low-salinity soil under sorghum planting (Fig. 4). The abundance of *Proteobacteria* was significantly higher in high-salinity soil than low-salinity soil, while *Actinobacteria* and *Acidobacteria* were much less abundant than *Proteobacteria*. These taxa may act as potential biomarkers for the sorghum response to salt concentration [24, 44].

Interactions between plant roots and soil microorganisms are critical for plant fitness in natural environments [41, 45]. Salinity stresses result in oxidative damage and even cell death in plants. Plants can adapt to the environment by regulating gene expression in roots and interacting with the soil bacteria by coordinating between the plant and soil bacteria for plant growth under abiotic stress [10, 43–48]. Many studies have shown that planting plants in soils with high salinity changes the composition of microbial communities [43]. In our study, planting sorghum and sorghum development in saline soil elicited shifts in bacterial community composition (Figs. 1, 2). These results indicated that there were interactions among salt, sorghum growth, and the soil bacteria.

Moreover, the sorghum variety GLZ showed better tolerance to high salinity stress, and the total phenol and total flavonoid contents in its root and rhizosphere soil during sorghum development were higher than those in the soils and roots of HN16.

Plants exude variable substances to create a diverse chemical environment. The composition of root exudates depends on the plant species, developmental stage, root traits, environmental conditions, nutrition, and soil type [10]. Exuded compounds have been shown to attract beneficial microorganisms and influence the structure and diversity of rhizosphere microbiomes that help plants adapt to their environment [49]. Phenolic compounds are important small signaling molecules exuded by plants [49–52] and are the most widely distributed secondary metabolites in plants as scavengers of excess O^2−^, H_2_O_2_, and ^1^O_2_ [53]. Their accumulation is thought to be essential for plants to adapt to a terrestrial environment, to be induced by abiotic stresses, and to be a hallmark of plant stress [50, 52–62]. Salt-tolerant species often accumulate more flavonoids and phenols than salt-sensitive species, suggesting a relationship between phenolic compounds and salt stress resistance [57]. Many studies have demonstrated the effect of phenolic compounds on the interactions between plants and microorganisms in the rhizosphere. For example, studies have found that flavonoids and small molecules help to establish a symbiotic relationship between rhizobacteria and plants [52], and some kinds of phenolic compounds, such as parabens, flavonols, and (iso) flavones, have a positive effect on spore germination and the mycelial growth of AM fungi [60]. Phenolic compounds exist widely and are essential components of active defense mechanisms in sorghum under biotic and abiotic stress [60–64]. Our results showed that phenol and flavonoid contents in rhizosphere soil were positively correlated with those in sorghum roots (Table S2), suggesting that sorghum can exude phenols into soil. Thus, a positive relationship between the phenol and flavonoid contents and sorghum resistance to salinity was also detected in our study. It was reasonable to suppose that the macrobiome communities and diversity were different between rhizosphere soils in the highly salt-tolerant GLZ and the salt-sensitive HN16 under salt stress. Although no significant difference in microbiome OTUs was detected between the two varieties by PERMANOVA, the interaction between salt concentration and sorghum genotype and development was significant. The high-tolerance sorghum genotype GLZ could mitigate the negative effects of salt stress by altering gene expression and dynamic root exudate chemistry to modify the soil microbiome to overcome salt stress (Fig. 5) [10, 43].

The variation in bacterial community composition according to salinity concentration, sorghum planting, sorghum growth, and genotype revealed in this study has implications for species sorting, with more salt-tolerant species replacing less salt-tolerant species in salinity soil cultivation, and for selecting a high salt tolerance indicator bacterial species.

Our data suggest that the presence of salt and sorghum result in changes to the bacterial community and diversity. Sorghum plants coordinate root bacterial communities to adapt to the soil environment to ensure their growth under stress conditions. The interaction among salinity, the root bacteria, plant growth, and the genotype could pave the way for technologies that modulate the root bacteria to increase crop adaptation to saline soil. Further studies are needed to have sight into the mechanism of these interactions.

## Conclusions

In this paper, we found that salt variability and planting were key factors in shifting bacterial diversity and community. Compared to bulk soils, soils under planting sorghum in different salt stress exhibited different shifts in a microbial community. Different types of sorghum with different levels of salt tolerance had different responses to salt stress by regulating root exudation. Soil bacterial community responses to salinity and exotic plants could potentially impact the microenvironment to help plants overcome outer stress and to promote sorghum growth. While this study observed bacterial responses to combined effects of salt and sorghum development, future studies are needed to understand the interaction among bacterial communities, salinity, sorghum growth and root exudates.

## Material and methods

### Plant materials

Two sorghum varieties (Gaoliangzhe ‘GLZ’ and Henong No.16 ‘HN16’) were used for all experiments. GLZ is a local variety with high salt tolerance, and HN16 is a seed variety and salt-susceptible.

### Soil preparation

The soil needed for this study was obtained from the Breeding Center of Hebei Agricultural University (E115°48′, N38°85′). The arable soil samples were obtained from 15 to 30 cm deep in the surface soil layer, and visible weeds, straw, and twigs were removed. The soil samples were air dried and sieved using a 2-mm mesh sieve for future experiments. The physiochemical properties of soil were examined before being added to the pot: pH 7.5, total nitrogen 1.00 g kg^− 1^, available phosphorous 20.1 mg kg^− 1^, and available potassium 170.9 mg kg^− 1^, organic content 9.75 g kg^− 1^. For the salt-treated soil group, the NaCl was added into soil as required and stirred well mixed with soil, the electrical conductivity was tested to determine the salt concentration. Four NaCl concentrations: 0% (Ck: S0), 0.3% (S3), 0.5% (S5) and 0.7% (S7). Sorghum seeds were grown in pre-sterilized plastic pots (34 cm diameter; 37 cm height). There was a matching tray for each pot to control moisture.

### Plant culture

Sorghum seeds were surface sterilized with 75% ethanol for 15 min and then 30 min in 1.2% hypochlorite, rinsed for 30 min with sterile distilled water, and dried at room temperature. Seeds were germinated on plastic trays overlaid with sterile paper in the dark for 2–3 d at 28 °C. After germination, three full grow nearly identical seedlings were transplanted into prepared soils and grown in a greenhouse (25–28 °C, with 16/8-h light/dark, 70% relative humidity). The soil water-holding capacity was kept at 70%. The exuded water was poured back into the pots in time to ensure salt concentration stability.

The experimental groups were (i) soils with different salt concentrations planted with sorghum (p: No. 1–24) and (ii) soils with different salt concentrations but without sorghum as controls (b: No. 25–28) (Table S3). Three biological replicates of each treatment were performed in a random arrangement. Three seedlings were planted in each pot, and plant heights were recorded at different stages. The plant and soil samples were collected at the initial (D0: no plant sample), jointing (D1), blooming (D2), and physiological maturity (D3) stages for physiological and microbial diversity analyses, respectively.

### Plant and rhizosphere soil sample collection

Plants that were healthy, grew uniformly, and had no visible pests were selected for soil sampling. First, a 10–20 cm soil profile was removed. Each pot was inverted to remove the soil and plants, and the sorghum roots were carefully dug up. The roots were shaken gently to remove the soil that did not adhere to the root surface. Then, the soil tightly adhered to the root surface (1–3 mm thick) was carefully collected with brushes and tweezers and defined as rhizosphere soil. Simultaneously, plant root samples were collected. After collection, the samples were immediately placed into sterile plastic bags in an ice box and then taken to the laboratory. The soil samples were passed through a 2-mm mesh sieve, and then they and the plant samples were stored at − 80 °C before processing.

### Bacterial diversity analysis

#### DNA extraction and PCR amplification

Microbial DNA was extracted from sorghum rhizosphere soil samples using the E.Z.N.A.® Soil DNA Kit (Omega Bio-tek, Norcross, GA, U.S.) according to the manufacturer’s protocols. The final DNA concentration and purification were determined using a NanoDrop 2000 UV-vis spectrophotometer (Thermo Scientific, Wilmington, USA), and the DNA quality was checked by 1% agarose gel electrophoresis. The V3-V4 hypervariable region of the bacterial 16S rRNA gene was amplified with primers 338F (5′- ACTCCTACGGGAGGCAGCAG-3′) and 806R (5′-GGACTACHVGGGTWTCTAAT-3′) [65] using a thermocycler PCR system (GeneAmp 9700, ABI, USA). The PCRs were conducted using the following program: 3 min of denaturation at 95 °C; 27 cycles of 30 s at 95 °C, 30 s for annealing at 55 °C, and 45 s for elongation at 72 °C; and a final extension at 72 °C for 10 min. PCRs were performed in triplicate in a 20-μL mixture containing 4 μL of 5 × FastPfu Buffer, 2 μL of 2.5 mM dNTPs, 0.8 μL of each primer (5 μM), 0.4 μL of FastPfu Polymerase (Abbexa, Cambridge, UK) and 10 ng of template DNA. The resulting PCR products were extracted from a 2% agarose gel and further purified using the AxyPrep DNA Gel Extraction Kit (Axygen Biosciences, Union City, CA, USA) and quantified using QuantiFluor™-ST (Promega, USA) according to the manufacturer’s protocol.

### Illumina MiSeq sequencing

Purified amplicons were pooled in equimolar amounts and paired-end sequenced (2 × 300) on an Illumina MiSeq platform (Illumina, San Diego, USA) according to the standard protocols by Majorbio Bio-Pharm Technology Co. Ltd. (Shanghai, China). The 16S rRNA gene fragment raw reads obtained from the sequencing company were deposited at the Genome Sequence Archive (GSA) under the project number PRJCA002781.

### Processing of sequencing data

Raw fastq files were quality-filtered by Trimmomatic and merged by FLASH with the following criteria. (i) The reads were truncated at any site receiving an average quality score < 20 over a 50-bp sliding window. (ii) Sequences with an overlap longer than 10 bp were merged according to their overlap with a mismatch of no more than 2 bp. (iii) Sequences of each sample were separated according to barcodes (exactly matching) and primers (allowing 2 nucleotide mismatches), and reads containing ambiguous bases were removed.

Plants height(PH, cm), main panicle length (MPL, cm), main panicle weight (MPW, g) and thousand grain weight (TGW, g) were measured at maturity.

### Determination of total phenol and total flavonoid contents

The roots were washed with fresh water, chopped into small pieces, and then oven-dried together with soil samples. The material was ground to a fine powder using an electric grinder. The total phenol content was determined by the Folin-Ciocalteu method [66]. A total of 0.1 mL sample was homogenized mixed with 2 mL of a 2% Na_2_CO_3_ solution freshly prepared, then vigorously and fully mixed on a vortex oscillator. After 5 min,100 ml of Foline–Ciocalteu reagent(1 N) were added to the mixture. After incubation at room temperature for 2 h, the reading of absorbance (SPECORD200Plus) is performed against a blank at 750 nm.A calibration curve was performed in parallel under the same operating conditions using gallic acid as a positive control.

A total of 0.1 mL sample was homogenized mixed with 2 mL of a 2% Na_2_CO_3_ solution pre-prepared, then vigorously and fully mixed on a vortex oscillator. Added 100 mL Folin-Ciocalteu reagent (1 N) to the aforementioned mixture. After incubation at room temperature for 2 h, the absorbance was read in quartz test tube at λ = 725 nm and performed against blank condition. The calculations were based on five-point standard curve dilutions of gallic acid (from 0 to 3 mg/mL in 50% methanol, R^2^ > 0.99). The results are expressed as mg gallic acid equivalent (GAE)/g sample, on a dry basis (db). All determinations were performed in triplicated and the data were reported as means ± SD.

The determination of total flavonoid content was performed according to the method of Zou et al. [67] with slight modifications. One milliliter of 5% sodium nitrite solution was added to 1 mL of total flavonoid extract, shaken well, and allowed to stand for 6 min in the dark. Then, 1 mL of 10% aluminum nitrate solution was added to it and shaken well. After 6 min in the dark, 10 mL of 4% sodium hydroxide solution was added and mixed well, diluted to 25 mL with deionized water, shaken, and allowed to stand for 10 min. The supernatant was absorbed, and its absorbance was measured at 510 nm. Catechi was used as standard compound for the quantification of total flavonoid. The preparation of the calibration curve was the same as the gallic acid. Calculated the results expressed as mg catechi equivalent per gram of dry extract (mg QE/100 g dry wt). All determinations were performed in triplicatea and the data were reported as means ± SD.

### Data analysis

Bacterial analysis was performed by rarefying the dataset to the lowest number of read counts by randomly selecting subsets of sequences. Plot bars were used to visualize taxonomic composition. Operational taxonomic units (OTUs) were clustered with a 97% similarity cutoff using UPARSE (version 7.1 http://drive5.com/uparse/) with a novel ‘greedy’ algorithm that performs chimera filtering and OTU clustering simultaneously. The taxonomy of each 16S rRNA gene sequence was analyzed by the RDP Classifier algorithm (http://rdp.cme.msu.edu/) against the Silva (SSU123) 16S rRNA database using a confidence threshold of 70%.

Statistical analyses were conducted using SPSS 20.0 (IBM, Chicago, USA) Analysis of variance and least significant difference (LSD) analysis were performed to test the significance of the salt effect on plant height, root exudates using SPSS 20.0.and R. Redundancy analysis was performed using the vegan library in R [68] to determine correlations of salt concentration, growth, variety, phenol content, and microbial communities. PERMANOVA (permutational multivariate analysis of variance) was performed using Bray-Curtis distances to determine the effect of variety, salt concentration and development on the bacterial diversity.

Pearson correlation analysis between different treatment and bacterial taxa was performed using the psych library in R. The R version (R Core Team) was used to perform statistical analysis and create graphs unless stated otherwise. Significant differences (*p* < 0.05) of bacterial gene copy numbers per gram of samples were analyzed using the Kruskal Wallis test. Analysis of the differential OTU abundance and taxa was performed using Wilcoxon rank sum tests based on OTUs or on phyla with mean relative abundance from each treatment.

## Supplementary Information

**Additional file 1: Figure S1.** The dominant phyla (average relative abundance > 1%) of root-associated bacteria based on 16S rRNA gene sequencing.

**Additional file 2: Figure S2.** Linear Discriminant Analysis.

**Additional file 3: Table S1.** Summary of samples sequences.

**Additional file 4: Table S2.** Correlation among the diversity index and phenolic compounds.

**Additional file 5: Table S3.** Treatment and sample name.

## Data Availability

All sequencing data associated with this study are deposited in GSA (http://bigd.big.ac.cn/gsa/s/5bkZedbF).

## Abbreviations

RDA: Redundancy analysis
ROC: Receiver operator characteristic curve
AUC: Area under the curve
LEfSe: LDA effect size
LDA: Linear discriminant analysis
