# Supplementary figures and images for: Changes in the root-associated bacteria of sorghum are driven by the combined effects of salt and sorghum development
Source: Environ Microbiome. 2021 Aug 11;16:14. doi: 10.1186/s40793-021-00383-0 (PMC8356455; doi:10.1186/s40793-021-00383-0)

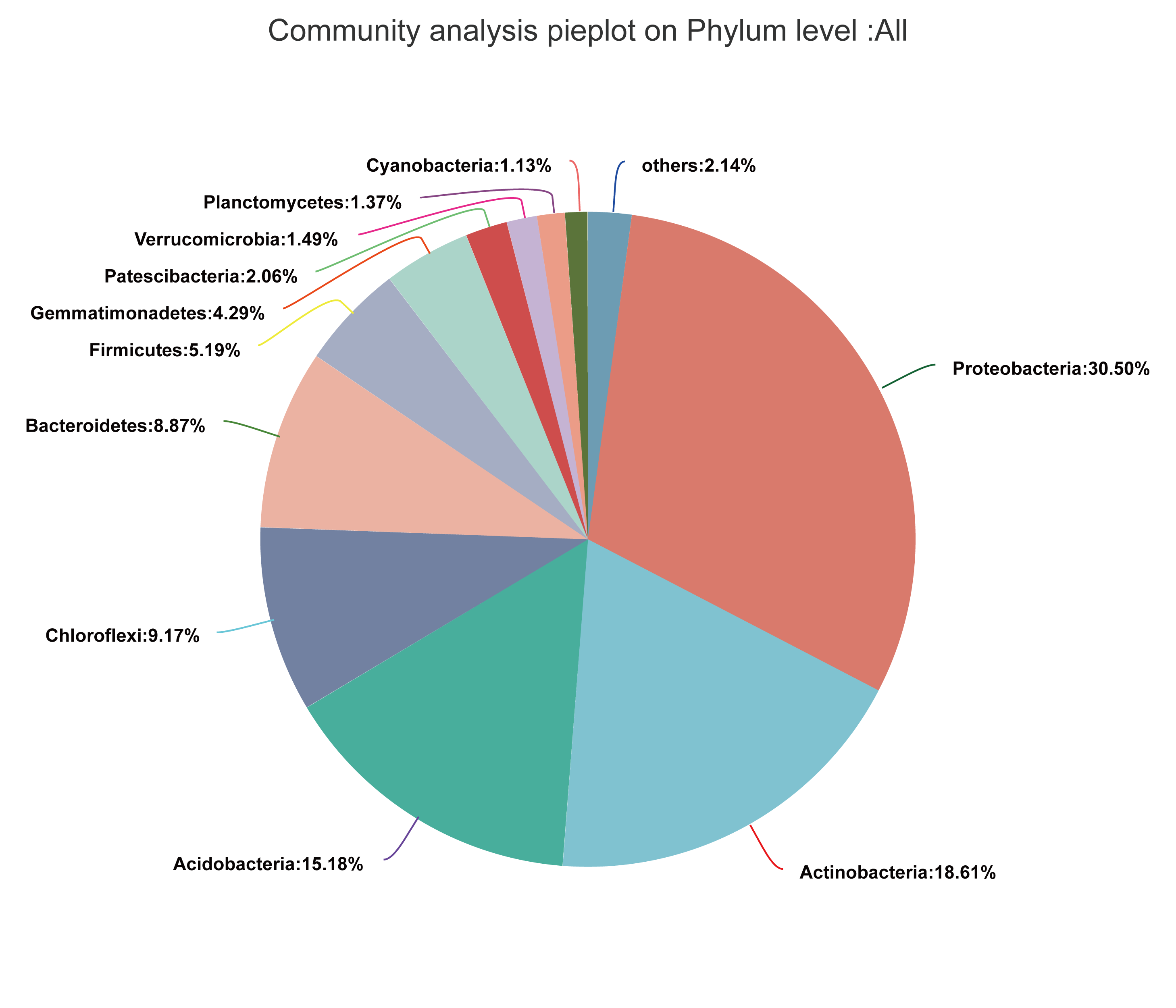

Supplement: Supplementary file 1 — Additional file 1: Figure S1. The dominant phyla (average relative abundance > 1%) of root-associated bacteria based on 16S rRNA gene sequencing. [file 40793_2021_383_MOESM1_ESM.tif]

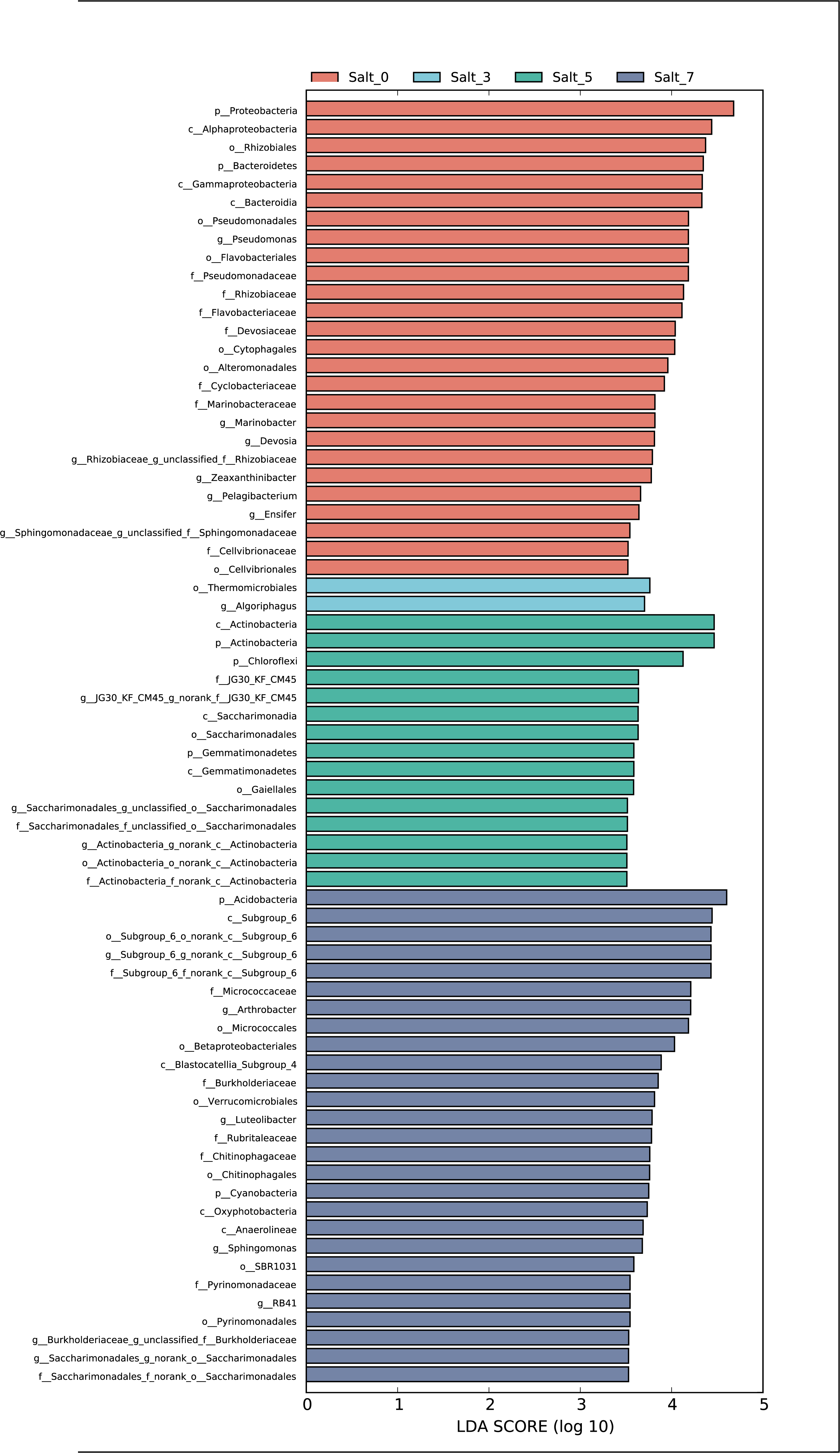

Supplement: Supplementary file 2 — Additional file 2: Figure S2. Linear Discriminant Analysis. [file 40793_2021_383_MOESM2_ESM.tif]
